# WaveMamba-YOLO: Combining frequency awareness and state-space modeling for defect localization

**DOI:** 10.1371/journal.pone.0344940

**Published:** 2026-03-20

**Authors:** Aping Ge, Yang Lv, Jun Huang

**Affiliations:** 1 Faculty of Intelligent Manufacturing, Wuhu Vocational Technical University, Wuhu, Anhui, China; 2 Yalong Intelligent Equipment Group Co., Ltd, Wenzhou, Zhejiang, China; 3 Faculty of Information Technology, City University of Malaysia, Kuala Lumpur, Malaysia; The University of British Columbia, AUSTRALIA

## Abstract

Steel surface defect detection is critical for ensuring the reliability and safety of automotive manufacturing. However, existing methods often suffer from high computational cost, weak sensitivity to fine textures, and limited adaptability to diverse defect scales. To address these challenges, we propose WaveMamba-YOLO, a real-time detection framework that integrates frequency-domain enhancement with efficient state-space modeling. The architecture introduces three key modules: (1) CHDWT, which combines Haar wavelet decomposition and residual learning to preserve structural details during downsampling; (2) GLaM, a global-local-aware Mamba module that couples large-kernel convolution with state-space modeling to capture long-range dependencies at linear complexity; and (3) LWGA, a lightweight group attention mechanism that adaptively attends to micro-, regular-, medium-, and large-scale defects. Experiments on the Severstal Steel Defect and NEU-DET datasets demonstrate that WaveMamba-YOLO achieves superior performance, reaching 51.70% mAP@0.5 and 58.60% precision on Severstal and 77.70% mAP@0.5 on NEU-DET, consistently surpassing mainstream lightweight detectors. These results confirm the effectiveness of WaveMamba-YOLO in balancing detection accuracy and efficiency, highlighting its potential for real-time industrial inspection.

## Introduction

Quality control is well recognized in the automotive manufacturing industry, where it plays a vital role in ensuring both product performance and safety. Moreover, it serves as a key indicator of the overall manufacturing process and technical capabilities across different sectors of the industry. Among the critical materials used in automotive production, steel is extensively employed in the construction of vehicle structures and components. The quality of steel processing has a direct impact on the structural stability and service life of the automobile. Defects in steel components can lead to severe economic consequences and may even pose safety risks to drivers and passengers. Traditionally, the detection of surface defects in steel components has relied heavily on manual visual inspection. However, this method suffers from several drawbacks, including high labor intensity, low efficiency, and strong dependence on the operator’s experience and subjective judgment. These limitations often result in inconsistent inspection outcomes, including missed or incorrect detections. To enhance the reliability of detection, researchers have proposed some product-specific NDT techniques, such as ECNT (Eddy Current Nondestructive Testing) based on the principle of electromagnetic induction [1], a technique based on the integration of microwave and thermographic NDT [2], and the use of structured light for 3D scanning and tracking [3]. However, these methods continue to encounter challenges in practical applications, including limited material adaptability, inadequate defect recognition capabilities, and suboptimal real-time performance. Consequently, they are unable to fully meet the current industrial demand for efficient and accurate inspection.

The advent of the Industry 4.0 paradigm [4] has accelerated the shift toward intelligent and automated manufacturing systems. Visual inspection, as a significant means of information perception and intelligent analysis, is witnessing a marked increase in its utilisation within industrial scenarios. This transformation is expected to significantly improve product quality management while driving innovation in traditional manufacturing models. In visual inspection, classical image processing techniques initially dominated defect detection tasks. These methods typically follow a two-stage process: feature extraction and discriminant analysis. The goal of feature extraction is to derive descriptive information from image data that highlights defects, thus improving the classifier’s recognition accuracy. Existing approaches can be broadly categorized as follows: (1) Statistical feature-based methods, such as grayscale histograms [5] and local binary patterns (LBP) [6]; (2) Structural feature-based methods that rely on geometric properties [7]; (3) Filter-based methods including Sobel [8], Canny [9], and Gabor [10] operators; and (4) Frequency domain methods such as wavelet [11] and Fourier transforms [12]. Once features are extracted, classifiers like support vector machines (SVM) [13], back-propagation neural networks (BP) [14], and k-nearest neighbors (KNN) [15] are employed for defect recognition. However, these methods rely heavily on handcrafted features, have limited adaptability to diverse defect types, and often lack robustness in complex industrial environments. Moreover, while they focus on defect classification, they struggle to provide precise spatial localization, which is crucial for defect analysis, traceability, and maintenance.

With the rise of deep learning, particularly convolutional neural networks (CNNs), industrial surface defect detection has seen substantial improvements. CNNs offer robust automatic feature extraction and generalization capabilities, allowing for high-accuracy detection even in complex environments. Currently, deep learning-based surface defect detection methods can be grouped into three categories based on task objectives: classification, detection, and segmentation. For classification tasks, researchers aim to improve accuracy and generalization. For example, Zhang et al. [16] proposed TL-ResNet50, a migration-enhanced ResNet-50 model that achieved 99.4% accuracy on the NEU-CLS dataset. Jeong et al. [17] introduced a hybrid-DC model combining ResNet-50 and Vision Transformer, which reached 99.44% validation accuracy by incorporating hybrid attention and global context modeling. Feng et al. [18] integrated FcaNet and the CBAM module into ResNet-50 for hot-rolled steel strip classification, improving defect recognition with 94.85% accuracy. Singh et al. [19] evaluated VGG19, GoogLeNet, DenseNet, and EfficientNet for defect classification in machined parts, identifying EfficientNet-b0 as the most promising model. In semiconductor inspection, López et al. [20] developed a ResNet50-based classifier that achieved strong results through image enhancement strategies addressing class imbalance. Although current classification methods have performed well under standard image conditions, their limitations are becoming increasingly apparent. Classification models usually assume that the image contains only one major defective region that has been accurately cropped and localized; however, in practice, surface defects are often variable in number, randomly located, and may be interfered with by complex backgrounds. In addition, the classification model lacks spatial localization capability, which makes it difficult to support defect tracking and subsequent processing. Therefore, a classification strategy that relies solely on image-level labels is difficult to meet the needs of online detection and intelligent feedback in industrial scenarios. To realize pixel-level automatic defect detection, researchers turn to semantic segmentation methods. Examples include Chen et al. [21] with an improved SegNet for crack detection, Pan et al. [22] with DAN-DeepLabv3 + using dual attention for defect localization, and U-Net variants with skip connections for multi-scale feature fusion. Yang et al. [23] proposed NDD-Net, replacing skip connections with an AFB module to boost feature discrimination, while Li et al. [24] incorporated a multi-scale attention mechanism into U-Net to improve crack segmentation. However, although these methods offer precise contour extraction, they involve high computational costs and are difficult to optimize for real-time industrial deployment [25,26].

In recent years, researchers have conducted a significant number of explorations on single-stage target detection frameworks to achieve a balance between detection efficiency and accuracy. Sylvic et al. [27] designed an industrial defect processing framework integrating detection, classification, and tracking, utilising RetinaNet CNN to localise defects and reduce the misdetection rate through inter-frame consistency. Cheng proposed [28] DEA RetinaNet, introducing differential channel attention and an adaptive spatial feature fusion module to improve the performance of steel surface defect detection. Su et al. [29] constructed a MOD-YOLO network to address the problem of the limited receptive field of the YOLO series. They introduced a receptive field optimisation mechanism in the infrastructure for crack detection. Yu et al. [30] proposed a bi-directional feature pyramid network (BiFPN) to replace YOLO. The BiFPN (Bilateral Feature Pyramid Network) has been utilised to substitute for the pyramid pooling module of YOLOv8, to optimise the efficacy of multi-scale feature fusion. Cao et al. [31] have developed a high-precision detection model by enhancing YOLOv5, achieving an accuracy of 74.1%. Fu [32] proposed the RCD-YOLO framework, which integrates the RE-CAF attention mechanism into the backbone to enhance defect feature representation, and employs the CARAFE lightweight up-sampling operator in the neck to improve cross-scale feature aggregation for crack detection. Zhao’s [33] design of the attention multiscale fusion module (AMFF) aims to enhance the robustness of defect recognition. The ADE-YOLO [34] approach integrates the attention mechanism with cavity convolution to effectively enhance multiscale feature expression. Conventional CNNs are deficient in their capacity to capture global context and are challenging to utilise for modelling long-range dependencies. Transformer architecture has been demonstrated to facilitate enhanced global modelling capability through the utilisation of the self-attention mechanism. This mechanism has been progressively employed in the context of defect detection. Wu et al. [35] integrated the Transformer and CBAM into the enhanced YOLOv5 architecture, achieving a detection accuracy of 74.8%. Yan et al. [36] proposed a multi-head attention mechanism based on SSD to enhance the detection of small targets. Guo et al. [37] developed MSFT-YOLO, which introduces the TRANS module in the backbone network and the detection head to improve the prediction capability of complex defects.

Despite the notable advantages of Transformer-based architectures in modeling long-range dependencies and capturing global context, their application in industrial surface defect detection still faces several critical challenges. First, their inherent self-attention mechanism, while powerful, incurs substantial computational overhead, making real-time deployment difficult, especially in high-throughput production lines. Second, Transformers tend to exhibit limited sensitivity to subtle edge variations and localized texture anomalies, which are essential for identifying fine-grained defects such as micro-cracks or surface scratches. These limitations restrict their effectiveness in scenarios that demand both precision and efficiency. In parallel, widely adopted modules such as the Spatial Pyramid Pooling-Fast (SPPF) in YOLO frameworks provide a degree of multi-scale perception, yet their fixed receptive field structures lack adaptability to the diverse and irregular defect patterns typically found in industrial steel surfaces. As a result, their generalization capability across different defect types and scales remains constrained. Furthermore, traditional downsampling methods—such as strided convolutions and max pooling—often discard high-frequency components during resolution reduction, leading to degraded edge continuity and insufficient preservation of fine texture details. This loss of critical information ultimately undermines the detector’s ability to localize and characterize minor surface anomalies with high fidelity.

To address these challenges, we propose WaveMamba-YOLO, a novel object detection framework tailored for high-precision surface defect inspection in complex industrial environments. Built upon the YOLO architecture, WaveMamba-YOLO introduces three structural innovations that jointly enhance global contextual modeling, spatial detail retention, and multi-scale adaptability:

1 **Direction-Aware Global Modeling via GLaM:** To address the difficulty of detecting fine-grained or elongated defects that are often obscured by complex backgrounds and overlooked during multi-scale feature fusion, we introduce the GLaM module into the neck. Unlike Transformer-based approaches that suffer from quadratic computational complexity when modeling long-range dependencies, GLaM adopts a channel-parallel structure that integrates local convolutional encoding with global state-space modeling based on Mamba and quad-directional Selective Scan (SS2D). This design enables the network to efficiently capture long-range dependencies and directional continuity with linear complexity, thereby enhancing its ability to distinguish subtle defect features from redundant background noise and improving overall detection robustness.
2 **Frequency-Enhanced Backbone with CHDWT:** Traditional downsampling operations (e.g., stride convolutions or pooling) often result in the loss of subtle edge or texture information critical for defect recognition. To address this, we propose the Channel-Enhanced Haar Discrete Wavelet Transform (CHDWT) module. This module fuses wavelet-based frequency-domain decomposition with residual semantic learning, enabling the network to retain both high-frequency structural cues and semantic integrity. As a result, it strengthens early-stage feature representations and enhances structural awareness in defect-prone regions.
3 **Multi-Scale Defect Perception with LWGA:** We introduce the Lightweight Group Attention (LWGA) module to address diverse defect scales and morphologies. It includes four specialized submodules:
  - GPA (Gate Point Attention): Targets micro-level defects such as fine scratches and pits.
  - RLA (Regular Local Attention): Captures geometric regularity in structured defects (e.g., cracks).
  - SMA (Sparse Medium-range Attention): Focuses on clustered or irregular mid-scale defects using sparse directional attention.
  - SGA (Sparse Global Attention): Efficiently models large-area surface anomalies, such as patches, inclusions, and rolled-in scale, by leveraging hybrid sparse attention strategies that balance contextual breadth with computational efficiency.

Together, these components enable WaveMamba-YOLO to achieve a balance between real-time efficiency and high-precision defect localization, offering a scalable solution for next-generation intelligent quality control systems in automotive steel production.

## Materials and methods

### Wave Mamba-YOLO

WaveMamba-YOLO is a novel object detection framework specifically designed to meet the demands of accurate, real-time surface defect detection in industrial environments. While retaining the foundational structure of YOLOv11 [38]—which includes a backbone for hierarchical feature extraction, a neck for multi-scale feature fusion, and multi-resolution detection heads—WaveMamba-YOLO introduces three critical architectural innovations aimed at enhancing its representational power, especially when faced with complex, fine-grained, and irregular steel surface defects.

**Fig 1:**
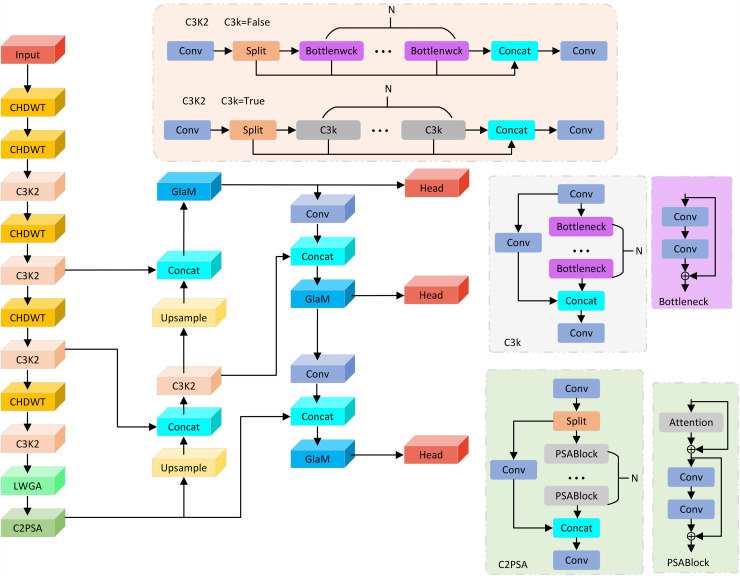
Architecture of the WaveMamba-YOLO.

As illustrated in Fig 1, we first redesign the backbone by embedding a series of Channel-Enhanced Haar Discrete Wavelet Transform (CHDWT) modules in place of conventional downsampling layers. By integrating frequency-domain decomposition with residual semantic fusion, CHDWT effectively preserves high-frequency edge and texture information that is typically degraded by standard strided convolutions. This design enhances structural integrity in early-stage features and significantly improves the network’s sensitivity to subtle surface anomalies.

To further address the difficulty of detecting small or elongated defects that may be overlooked due to limited contextual perception during feature aggregation, we introduce the GLaM module into the neck. GLaM adopts a channel-parallelized architecture, where a local branch based on CBS (Convolution–BatchNorm–SiLU) blocks encodes fine structural details, while a global branch leverages the Mamba state-space mechanism together with Selective Scan to model long-range dependencies and directional continuity. This cooperative design enables the network to jointly capture local variations and global spatial structures with linear computational complexity, thereby enhancing robustness against complex and anisotropic defect patterns.

In addition, we replace the traditional SPPF module with a more adaptive and scale-aware LWGA module. LWGA comprises four parallel attention branches—GPA, RLA, SMA, and SGA—each specialized for defect patterns at different spatial scales. This targeted attention mechanism enables accurate characterization of micro-defects, regular cracks, irregular clustered textures, and large-area anomalies, ensuring reliable discrimination across a wide spectrum of defect types and sizes.

Together, the CHDWT, GLaM, and LWGA modules construct a compact yet powerful architecture that achieves enhanced defect representation while maintaining real-time efficiency. Consequently, WaveMamba-YOLO provides a practical and scalable solution for high-resolution automated surface inspection in industrial steel manufacturing.

### Global-local-aware mamba

**Fig 2:**
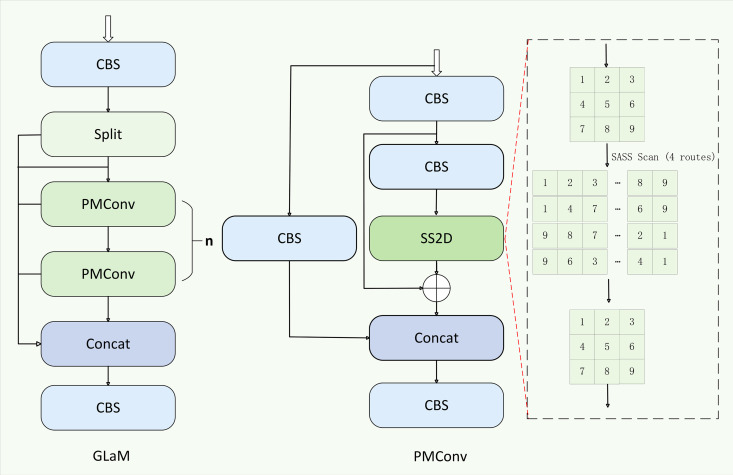
GLaM network structure diagram.

In steel surface defect detection, targets such as small-scale flaws, weakly textured anomalies, and fine-grained structures (e.g., micro-cracks or edge-like pits) are often obscured by complex background textures or noise patterns, leading to missed detections. Although the neck in YOLO-based detectors is designed to aggregate multi-scale features, it typically relies on shallow convolutional fusion, which cannot model long-range dependencies, global structural continuity, and directional context. As a result, the fused features in this region are often dominated by redundant background information and fail to capture the subtle cues necessary for detecting fine or irregular defects. To address these limitations, we introduce a state-space modeling mechanism into the neck by integrating the Mamba [39] architecture, which supports efficient long-range dependency modeling with linear complexity. Specifically, we employ a quad-directional selective scanning strategy to extract spatial sequences along multiple orientations, enabling the network to link local defect cues with broader structural patterns. In addition, Mamba’s gated design effectively filters out repetitive background textures by learning typical distribution patterns, thus enhancing the contrast between defective and non-defective regions. To this end, we propose GLaM, a multi-branch feature modeling module that combines parallel local processing with efficient global state-space modeling. This hybrid design captures both local texture transitions and global structural coherence, which are crucial for identifying complex and scale-diverse defects, including cracks, scratches, and delaminations. As shown in Fig 2, GLaM adopts a channel-parallelized architecture. The input feature map is first processed by a CBS block (Convolution–BatchNorm–SiLU) for normalization and activation. It is then split along the channel dimension into N parallel branches. Each branch is fed into a PMConv block, which contains two parallel paths: a standard convolutional path for local feature extraction and a state-space path for global modeling via Selective Scan (SS2D).

**Fig 3:**
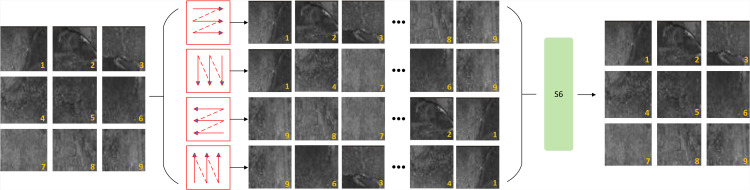
Quad-Directional 2D Selective Scanning (SS2D) Trajectories in the PMConv Module’s State-Space Path.


The PMConv module employs a dual-path architecture that synergistically integrates local convolutional processing with global state-space modeling. In its local path, a standard convolutional layer extracts fine-grained spatial features (e.g., micro-crack edges). In the state-space path, features first undergo embedding via two CBS (Convolution-BatchNorm-SiLU) layers, followed by quad-directional 2D Selective Scanning (SS2D) as illustrated in Fig 3—which is a core component of the PMConv module’s state-space path: decomposing the feature map into sequences along four distinct trajectories: top-left to bottom-right, top-right to bottom-left, bottom-left to top-right, and bottom-right to top-left (visualized by the red trajectory diagrams in Fig 3). Each directional sequence is processed independently by the S6 block:

$$y_{s}=S6(x_{s}),\;s\in \left\{\to ,\leftarrow ,↓,↑\right\},$$ (1)

The outputs from the four directions are reshaped and fused to produce a globally-aware representation. A residual connection is added for stable optimization:


$$Y_{Mamba}=SS2D(CBS(CBS(X)))+CBS(X),$$ (2)

Finally, the outputs of the local convolutional path $Y_{CBS}$ and the Mamba-based path $Y_{Mamba}$ are concatenated along the channel dimension and compressed through a final CBS operation:


$$Y=CBS(Concat(Y_{CBS},Y_{Mamba})),$$ (3)

The GLaM module is designed to capture complementary features by combining local detail perception with global structural modeling. While the convolutional branch focuses on extracting fine-grained textures essential for identifying small or edge-localized defects, the Mamba-based branch models long-range spatial dependencies and directional continuity. This integration enables the network to distinguish subtle defect patterns from complex backgrounds more effectively, resulting in improved detection precision and robustness across varying defect scales and morphologies, all while maintaining low computational overhead.

### Channel-enhanced Haar discrete wavelet transform

To enhance feature representation and preserve structural details on defect-prone steel surfaces, we propose a novel downsampling module named CHDWT. Unlike traditional stride convolution or pooling—which often discards fine-grained features—CHDWT leverages the frequency-domain decomposition capability of the Haar wavelet transform to retain texture, edge, and detail information critical for defect recognition. By combining wavelet-based structural priors with learnable convolutional processing, CHDWT achieves more informative and defect-sensitive feature encoding.

**Fig 4:**
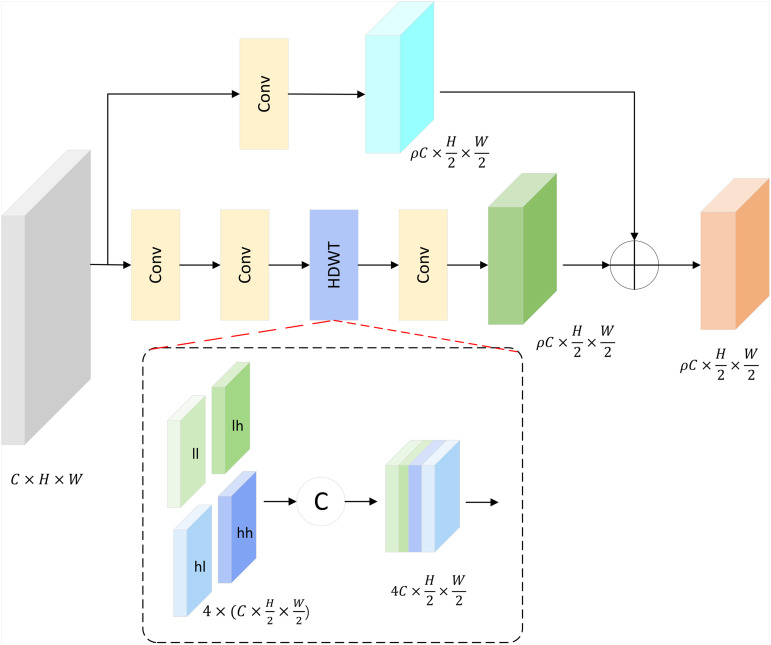
CHDWT network structure diagram.

Surface defects like cracks, inclusions, and pits usually present as local texture disturbances and abrupt edge transitions, subtle patterns that are easily blurred in conventional downsampling processes. To tackle this, CHDWT employs a dual-branch design where the wavelet prior branch uses Haar Discrete Wavelet Transform (HDWT) [40] to decompose input features into frequency-specific subbands, capturing horizontal, vertical, and diagonal gradients that accentuate structural changes from defects, while the convolutional branch carries out downsampling and boosts robustness against local intensity variations. By fusing the outputs of both branches, CHDWT efficiently maintains edge sharpness and fine texture details, enhancing the network’s sensitivity to defect-specific features, as shown in Fig 4.

Given an input feature map $X\in {\mathbb{R}}^{C\times H\times W}$, CHDWT outputs a downsampled representation $Y\in {\mathbb{R}}^{\rho C\times \frac{H}{2}\times \frac{W}{2}}$, where *ρ* denotes the channel expansion ratio. The module consists of the following components:

(a) **Wavelet Prior Branch**

This branch applies Haar DWT to extract frequency-aware features. Specifically, the HDWT decomposes the input into four subbands:


$$X_{ll},X_{lh},X_{hl},X_{hh}=HDWT(X)$$ (4)

Each subband captures distinct frequency responses, $X_{ll}$: low-frequency (global structure), $X_{lh},X_{hl},X_{hh}$: high-frequency details (vertical, horizontal, diagonal). The subbands are concatenated along the channel dimension:


$$X_{w}=\text{Concat}(X_{ll},X_{lh},X_{hl},X_{hh})\in R^{\frac{4C\times H}{2}\times \frac{W}{2}}$$ (5)

Followed by a convolution for channel compression:


$$Y_{w}=f_{\theta_{1}}(X_{w})Y_{w}\in R^{\frac{\rho C\times H}{2}\times \frac{W}{2}}$$ (6)

(b) **Residual Semantic Branch**

A separate convolutional path performs strided downsampling:


$$Y_{r}=f_{\theta_{2}}^{\left(s=2\right)}(X)\;Y_{r}\in R^{\frac{\rho C\times H}{2}\times \frac{W}{2}}$$ (7)

This branch captures spatial patterns and enriches cross-channel interactions that wavelet decomposition lacks.

(c) **Feature Fusion**

Finally, the outputs from the two branches are aggregated using element-wise addition:


$$Y=Y_{r}+Y_{w}$$ (8)

This residual fusion enables CHDWT to retain both structural priors from wavelet decomposition and semantic consistency from learned representations.

### Lightweight group attention

**Fig 5:**
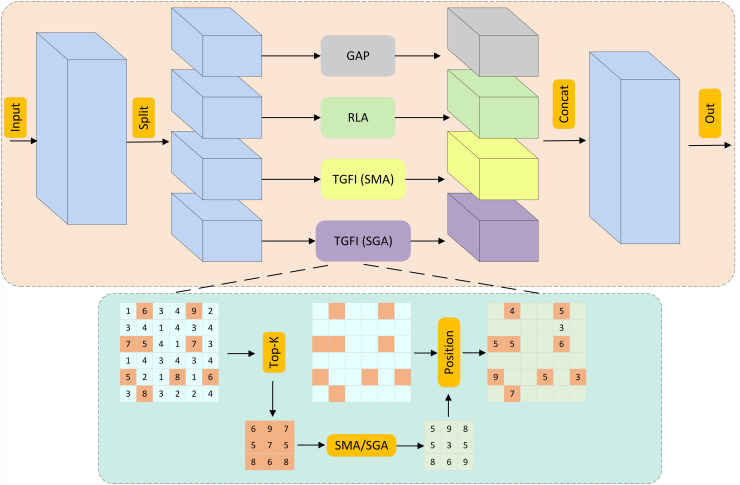
LWGA network structure diagram.

In standard YOLO architectures, the Spatial Pyramid Pooling – Fast (SPPF) module is widely used in the backbone to enhance the receptive field and capture multi-scale information. However, in steel surface defect detection, SPPF exhibits significant limitations. Its fixed pooling kernels and isotropic aggregation mechanisms are insufficient to adaptively handle the wide variability in defect scales, shapes, and spatial distributions. To overcome these limitations, we introduce the LWGA [41] module as an effective replacement for SPPF in the backbone. LWGA introduces scale-specific attention modeling through four specialized submodules, each tailored to different types of defect patterns. By focusing on different defect scales through lightweight attention mechanisms, LWGA effectively improves the detection of small details, regular structures, and large surface anomalies. As shown in Fig 5, the input feature map $X\in {\mathbb{R}}^{H\times W\times C}$ is partitioned into four parallel sub-features $\left\{X_{1},X_{2},X_{3},X_{4}\right\}$, where $X_{i}\in {\mathbb{R}}^{H\times W\times 4C}$. Each sub-feature is processed by specialized attention mechanisms to address the diversity of defect scales, morphologies, and low-contrast characteristics in industrial scenarios. The outputs are fused through lightweight operations to balance precision and computational efficiency.

The Gate Point Attention (GPA) submodule is specialized for detecting micro-defects such as sub-pixel pits and faint scratches. By applying a 1×1 convolution to expand channel dimensions, followed by a GELU activation and a Sigmoid-generated attention map *A1*, GPA refines features through R1=X1+A1⊙X1. This mechanism suppresses homogeneous background regions while amplifying high-frequency components associated with subtle defects, effectively enhancing local contrast for micro-anomalies.

For structured defects like linear cracks and edge burrs, the Regular Local Attention (RLA) submodule leverages a 3×3 depthwise convolution combined with batch normalization (BN) and ReLU activation. This design preserves the geometric regularity of defects in *R2*, ensuring precise localization and morphological analysis critical for industrial quality control.

Irregular mid-scale defects such as clustered scuffs and distributed corrosion are addressed by the Sparse Medium-range Attention (SMA). SMA incorporates a Top-k Global Feature Interaction (TGFI) mechanism that downsamples *X3* by retaining only the Top-30% salient features, followed by sparse attention computation across four directional paths (horizontal, vertical, and $\pm 45^{\circ }$ diagonals) with a span L=9. Positional encoding $L_{p}$ restores spatial relationships, enabling efficient capture of defect clusters in R3=A3⊙X3 without dense computational overhead.

The Sparse Global Attention (SGA) submodule adaptively processes large-area defects like stains and rolling marks through a dual-mode architecture. In shallow layers (Stages 1–2), dilated convolution (K = 5, D = 2) mimics global context aggregation, while deeper layers (Stages 3–4) employ multi-head self-attention (2 heads) with TGFI-based dimensionality reduction (Top-40% features). The residual output R4 integrates long-range dependencies while minimizing FLOPs, ensuring robust defect characterization across varying scales. These submodules collectively enable the LWGA to balance precision and computational efficiency, making it suitable for real-time steel surface inspection systems requiring both fine-grained defect detection and industrial-grade throughput.

## Results

### Experimental setup

#### Datasets.

**Fig 6:**
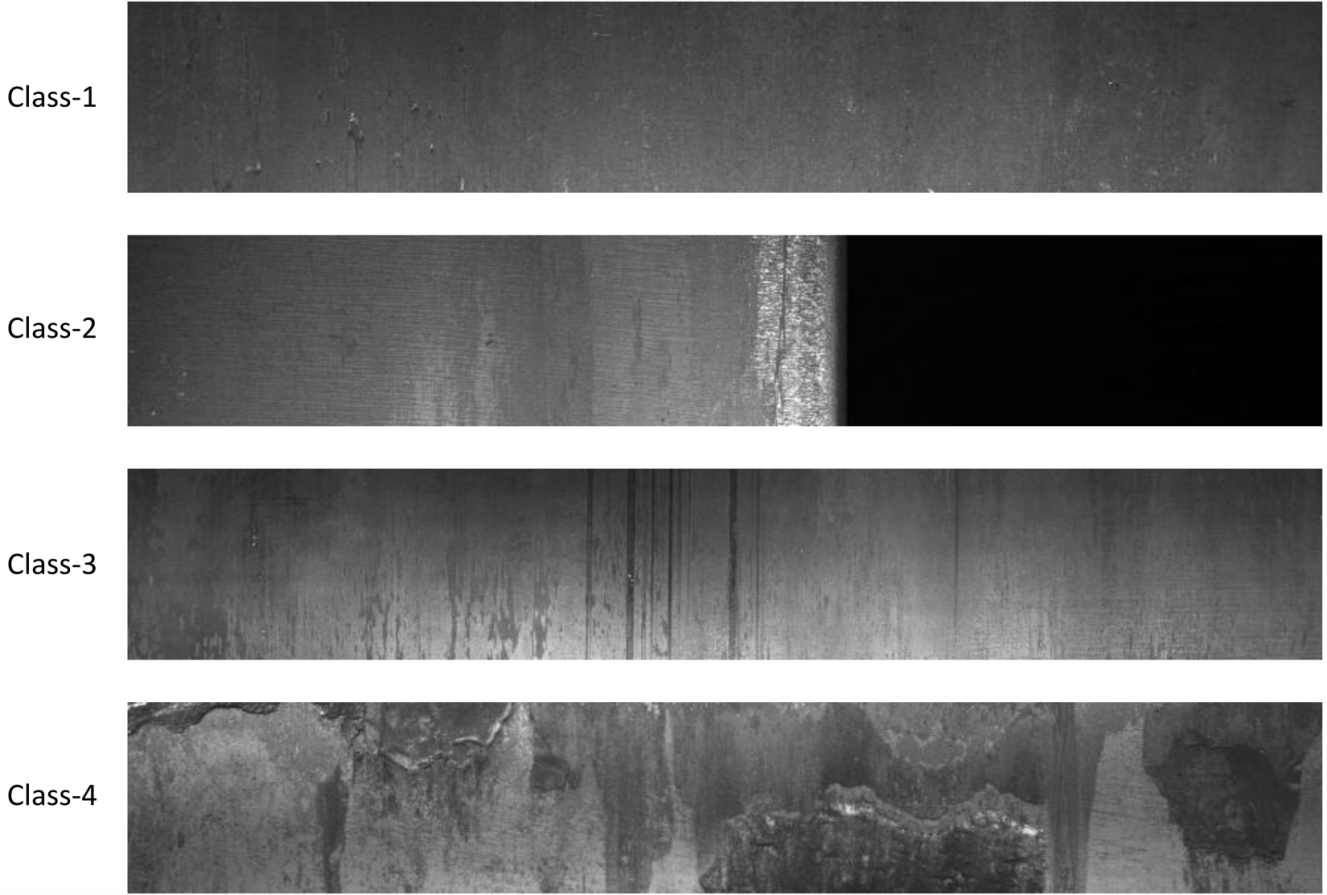
Example Diagram of Severstal Steel Defect Types.

The Severstal Steel Defect Detection dataset [42], released by leading steel manufacturer Severstal, is a high-resolution industrial benchmark dataset featuring grayscale images of steel surfaces captured during production, each potentially containing one or more defect types categorized into four classes: Class-1 (Spots/patches), Class-2 (Scratches), Class-3 (Surface Defects like inclusions and rolled-in scale), and Class-4 (Other miscellaneous or uncategorized anomalies). With a resolution of 1600×256 pixels, the dataset provides fine-grained details essential for precise defect localization and was constructed through expert annotation. Its diverse defect types, combined with challenges like imbalanced class distribution and subtle visual variations, make it a valuable resource for developing and evaluating robust defect detection algorithms in real-world industrial settings. As shown in Fig 6.

**Fig 7:**
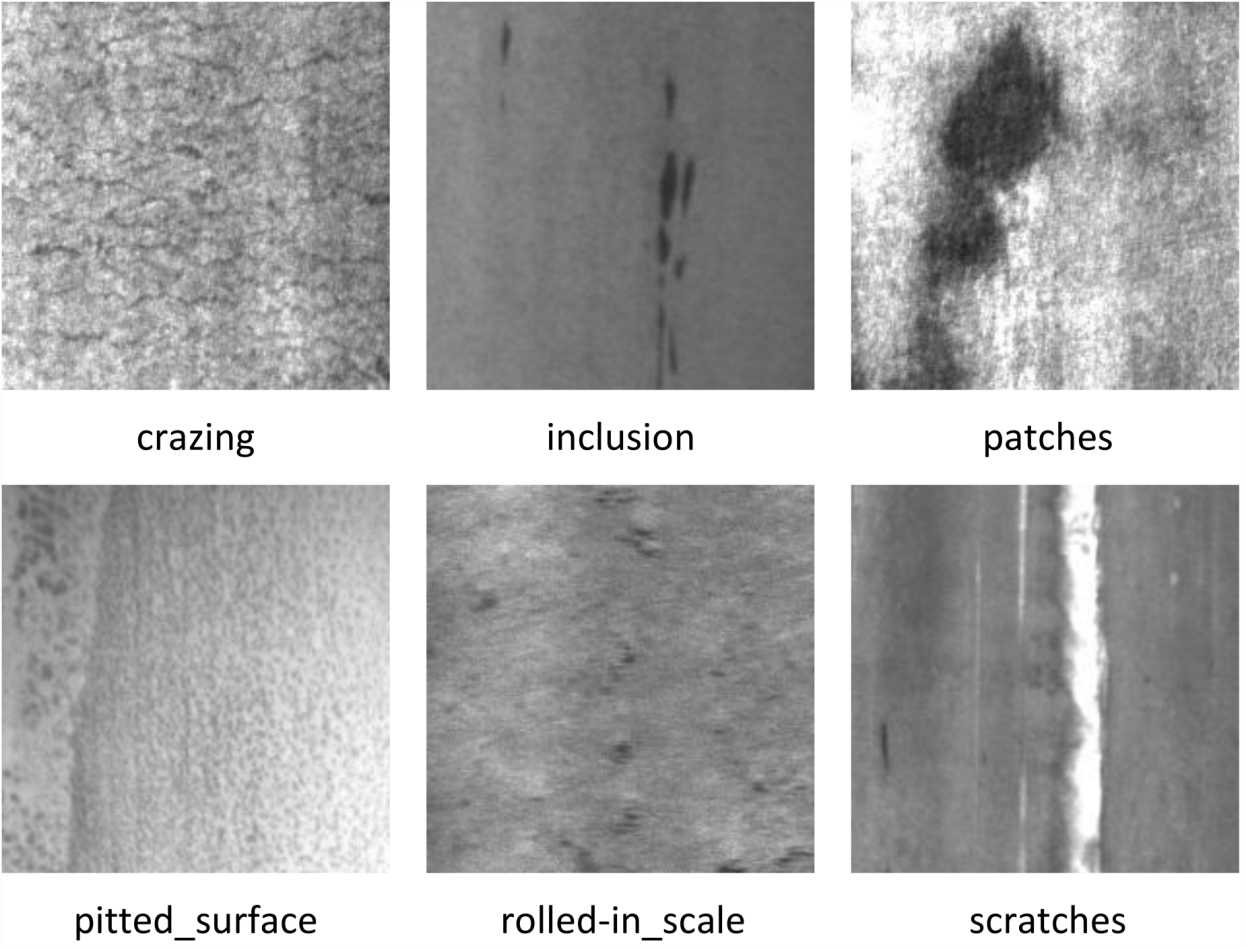
NEU-DET Dataset Defect Types Illustration.

The NEU-DET dataset [43] is a widely used benchmark for surface defect detection in industrial scenarios. It contains a total of 1,800 grayscale images with a fixed resolution of 200 × 200 pixels, collected from hot-rolled steel strips under realistic production conditions. As shown in Fig 7, the dataset is divided into six typical defect categories, namely crazing, inclusion, patches, pitted surface, rolled-in scale, and scratches, with each category comprising 300 samples. All images are manually annotated with bounding boxes to indicate defect locations, enabling its application in both classification and object detection tasks. Due to its balanced category distribution, standardized size, and clear defect patterns, NEU-DET has become a standard benchmark for evaluating defect detection algorithms.

**Fig 8:**
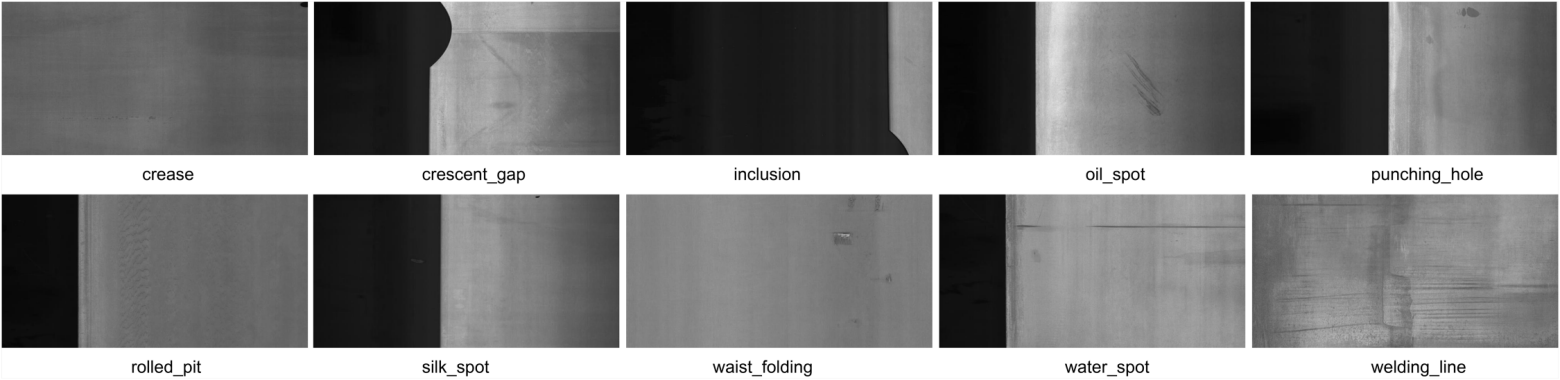
GC10-DET Dataset Defect Types Illustration.

The GC10-DET dataset [44] is a practical industrial benchmark derived from real steel sheet production, encompassing 3,570 grayscale images and ten distinct defect categories. As illustrated in Fig 8, the dataset covers a wide range of defect appearances, including low-contrast water spots and oil spots, as well as morphologically diverse silk spots and waist folds. All images are annotated with precise bounding boxes and reflect authentic variations encountered in industrial environments.

**Fig 9:**
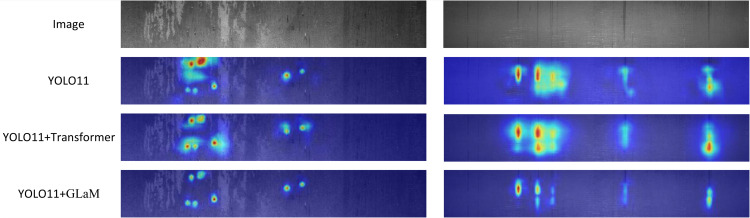
Class Distribution of the GC10-DET Dataset.

It is worth noting that GC10-DET exhibits a noticeable class imbalance among defect categories. As visualized in the class distribution histogram in Fig 9, some defect types are represented by a relatively large number of samples, whereas others occur far less frequently, resulting in a long-tailed distribution. This imbalance reflects the natural occurrence frequency of defects in real-world steel production and increases the difficulty of achieving stable performance across all categories.

### Evaluation indicators

In the evaluation of target detection models, the following four key metrics are frequently employed to gauge the model’s performance with regard to detection precision and recall capability:

1 **mAP50**

mAP50 is defined as the mean Average Precision (AP, Average Precision) for each category at an IoU (Intersection Over Union) threshold of 0.5. Intersection over Union (IoU) is utilised to quantify the extent of overlap between the predicted and actual frames. A prediction is deemed correct when IoU≥0.5. This metric is one of the most common accuracy evaluation metrics for detection tasks, and it is more lenient and suitable for evaluating the model’s ability to roughly detect the target position.


$$mAP@0.5=\frac{1}{N}\sum_{i=1}^{N}AP_{i}^{IoU=0.5}$$ (9)

Where *N* is the total number of categories, and $AP_{i}$ denotes the average accuracy of the *i*-th category under the condition of IoU=0.5. This metric measures the overall detection ability of the model under the condition of IoU≥0.5 between the target prediction frame and the true frame.

2 **mAP50:95**

The mean of the APs calculated at distinct IoU thresholds (from 0.5 to 0.95 in increments of 0.05) is denoted mAP50:95. This constitutes a more rigorous and comprehensive evaluation criterion, designed to assess the overall detection ability of the model under varying matching accuracy requirements. This approach is considered a superior metric for evaluating the generalisation capability and accuracy robustness of the model.


$$mAP@0.5:0.95=\frac{1}{10N}\sum_{k=0}^{9}\sum_{i=1}^{N}AP_{i}^{IoU=0.5+0.05k}$$ (10)

3 **Precision**

Precision is defined as the proportion of truly positive samples among all detection results that are judged as “positive samples” by the model. The formula is as follows:


$$\text{Precision}=\frac{TP}{TP+FP}$$ (11)

4 **Recall**

The recall is defined as the proportion of true positive samples that are correctly identified by the model. It is the measure of the model’s ability to recognise all true targets. The formula is as follows:


$$\text{Recall}=\frac{TP}{TP+FN}$$ (12)

where FN is the number of positive samples that were missed for detection. Higher recall means fewer missed detections

### Experimental environment

All experiments were conducted using the PyTorch deep learning framework. The primary training and evaluation were performed on a workstation equipped with an NVIDIA RTX 3090 GPU and an Intel Core i7-12700KF processor. Two system environments were used during the study: Windows 11 and Ubuntu 20.04 LTS, each configured with Python 3.10, CUDA 11.8, and cuDNN 8.6. The training process adopted an initial learning rate of 0.0001 and a batch size of 64. Each model was trained for 300 epochs. During training, an IoU threshold of 0.6 was applied to determine positive samples for object detection tasks. This configuration provides a balance between computational efficiency and model performance, enabling robust and reproducible results across platforms.

### Comparison of neck structures: GLaM vs. transformer

To evaluate the effectiveness of the proposed GLaM module, we conducted comparative experiments based on the YOLOv11n baseline. Specifically, we implemented and compared three variants: (1) the original YOLOv11n, (2) a Transformer-enhanced YOLOv11n [45], and (3) the proposed GLaM-YOLOv11n, which incorporates the GLaM module. All experiments were conducted with an input resolution of 640×640, and the evaluation metrics included mAP50, mAP50-95, Recall, Precision, and model parameters.

As shown in Table 1, the Transformer-YOLOv11n variant achieves a moderate improvement in detection accuracy, with mAP50 increasing by 0.6% compared to the baseline. However, it also introduces a significant increase in parameter count (from 2.58M to 3.25M) and a decrease in precision. This suggests that while the Transformer enhances global feature modeling, it may also lead to overfitting or noisy attention in small or texture-rich defect regions. In contrast, the proposed GLaM-YOLOv11n outperforms both the baseline and the Transformer-enhanced variant in all key metrics. It achieves the highest mAP50 (50.6%) and mAP50-95 (23.4%), along with the best precision (57.0%), while maintaining a lightweight structure (2.78M parameters). These results indicate that the GLaM module offers a more efficient and defect-sensitive alternative to Transformer-based designs. The superior performance of GLaM-YOLOv11n can be attributed to its channel-parallel architecture, which combines large-kernel convolutional encoding with Mamba-based state-space modeling. Unlike Transformer-based methods that rely on a quadratic self-attention mechanism, GLaM achieves linear computational complexity, making it more suitable for real-time applications. At the same time, the state-space design in GLaM offers strong global modeling capabilities while avoiding noise sensitivity often observed with Transformers in texture-rich or small-defect scenarios. Furthermore, the Selective Scan (SS2D) mechanism enables GLaM to model directional continuity along multiple spatial orientations, enhancing its ability to detect elongated, anisotropic, or weakly textured defects. This contributes to more stable and accurate feature representation, especially in the presence of complex backgrounds.

**Table 1:** Comparison Results for Three Different Necks on the Defect Dataset.

Method	Size	mAP50	mAP50-95	Recall	Precision	parameters
YOLOv11n	640 x 640	49.50	22.70	48.80	55.20	2.58
Transformer-YOLOv11n [45]	640 x 640	50.10	22.90	50.20	52.20	3.25
GLaM-YOLOv11n	640 x 640	50.60	23.40	49.30	57.00	2.78

**Fig 10:**
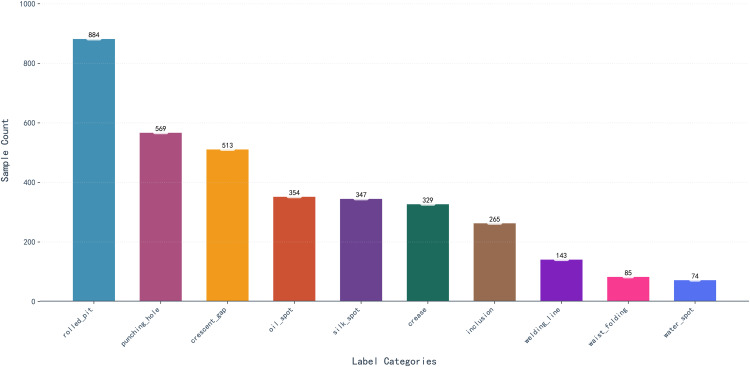
Comparison results of thermogram visualization of three different necks.

As shown in the heatmaps of Fig 10, the baseline YOLOv11n model has difficulty distinguishing defect regions from the background, often generating indistinct and overlapping outputs that merge multiple objects. This reflects its limited capacity to separate foreground anomalies from complex textures, particularly for closely clustered or low-contrast defects. The Transformer-enhanced YOLOv11n partially improves foreground-background discrimination via its global attention mechanism, but still exhibits diffuse responses and misactivation in uniform or noisy areas. In contrast, the proposed GLaM-YOLOv11n achieves notably clearer and more precise outputs. Defect regions are accurately outlined with sharp boundaries and minimal background interference. This improvement stems from GLaM’s channel-parallel architecture, which integrates local large-kernel convolution for capturing fine textures and Mamba-based state-space modeling with directional Selective Scan (SS2D) to maintain spatial continuity. These mechanisms collectively enhance the model’s ability to distinguish targets from complex industrial backgrounds, significantly boosting its localization performance in real-world defect detection scenarios.

### Comparative experiments on multi-scale modules

**Fig 11:**
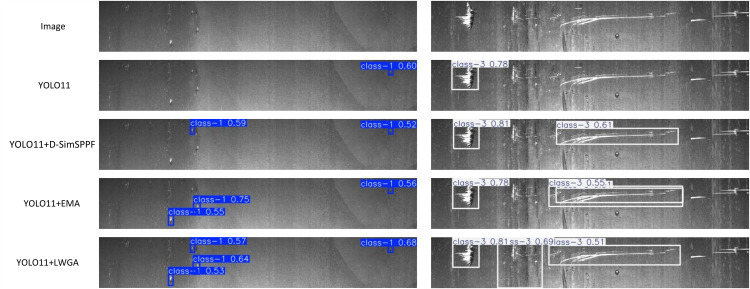
Radar Chart Comparison of YOLOv11n and YOLOv11n+CHDWT Across Multiple Metrics.

To evaluate the impact of frequency-domain enhanced downsampling on steel surface defect detection, we integrated the proposed CHDWT module into the YOLOv11n baseline for comparative experiments. Traditional downsampling operations (e.g., strided convolution, pooling) often damage structural integrity and weaken feature expressiveness—especially for fine-grained defects—whereas CHDWT combines the structural decomposition ability of Haar wavelet transform with channel-level residual modeling, aiming to preserve edge information while enhancing semantic feature encoding. Fig 11 presents a radar chart comparing YOLOv11n and YOLOv11n+CHDWT across four key metrics (mAP50, mAP50–95, Recall, Precision): integrating CHDWT brings consistent improvements, with mAP50 rising from 49.5% to 50.5%, mAP50–95 from 22.7% to 23.5% (boosting detection accuracy for both obvious and subtle defects), Recall from 48.8% to 49.6% (strengthening true positive identification, particularly for low-contrast or ambiguous defects), and a slight increase in Precision (from 55.2% to 56.1%, reducing false positives and improving localization reliability). Architecturally, the CHDWT module has two complementary branches: the wavelet prior branch uses Haar Discrete Wavelet Transform to decompose input features into directional subbands (capturing structural cues like horizontal/vertical gradients), while the residual semantic branch leverages learnable strided convolutions to model cross-channel semantic dependencies and maintain resolution consistency; features from both branches are fused to form a frequency-aware, semantically rich representation, preserving structural details in early layers and enhancing downstream detection performance.

### Comparative experiments on multi-scale modules

To further validate the effectiveness of the proposed LWGA module, we conduct comparative experiments against two representative Multi-scale modules adopted from existing literature: D-SimSPPF [46] from YOLOv7 and EMA [47] from YOLOv8. These modules are integrated into the YOLOv11n baseline for a fair comparison under identical experimental settings.

As illustrated in the Table 2, LWGA-YOLOv11n outperforms all compared variants, achieving the highest detection accuracy with a mAP50 of 51.2% and mAP50-95 of 23.3%, alongside the best precision (57.0%) with only 2.77M parameters. In contrast, the D-SimSPPF-enhanced model yields only marginal improvements over the baseline, indicating that simple multi-scale pooling, while lightweight, lacks the adaptivity required for handling diverse and irregular industrial defects. The EMA-based variant demonstrates slightly better performance in mAP50-95 (22.8%) due to its global and local attention fusion, but it introduces a higher parameter cost (2.89M) and does not surpass LWGA in any primary metric.

**Table 2:** Comparative results of three different multi-scale modules on steel defect datasets.

Method	Size	mAP50	mAP50-95	Recall	Precision	parameters
YOLOv11n	640 x 640	49.50	22.70	48.80	55.20	2.58
YOLOv11n+D-SimSPPE [46]	640 x 640	49.60	21.90	49.00	56.50	2.56
YOLOv11n+EMA [47]	640 x 640	49.90	22.80	48.30	55.70	2.89
YOLOv11n+LWGA	640 x 640	51.20	23.30	49.30	57.00	2.77

**Fig 12:**
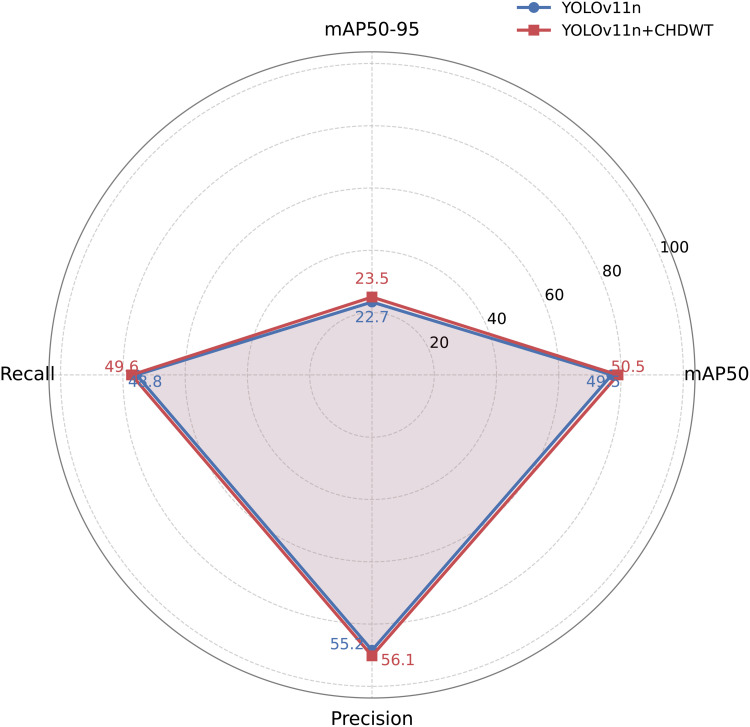
Comparison for Multi-scale Models.

To better illustrate the qualitative differences, Fig 12 presents visual comparisons of detection results across different models. The proposed LWGA-YOLOv11n shows superior localization capability and robustness across a wide range of defect types, including small-scale pits, narrow cracks, and large corrosion areas. Compared to other models, LWGA generates more accurate bounding boxes with higher confidence scores, especially in challenging scenarios involving low contrast or densely clustered anomalies. Notably, the D-SimSPPF and EMA-based variants often miss fine defects or produce imprecise boxes, highlighting their limitations in generalizing to complex surface textures. These results validate that the proposed LWGA module, with its four specialized submodules—GPA, RLA, SMA, and SGA—provides an effective multi-scale attention mechanism. By tailoring attention to specific defect morphologies, LWGA enhances feature discrimination and spatial adaptability across a broad range of defect types, all while maintaining model efficiency. This makes it particularly well-suited for real-time, high-resolution industrial defect inspection scenarios.

### Ablation study under different lighting conditions

To evaluate the contributions of the proposed modules, ablation experiments were conducted on the Severstal Steel Defect dataset using YOLOv11n as the baseline. For clarity, CHDWT, GLaM, and LWGA are denoted as C, G, and L, respectively. Experiments were performed under four representative industrial illumination conditions, including normal, low-light, over-light, and uneven illumination. Detection performance was evaluated using mAP_50_, while inference speed (FPS) and parameter count were reported to assess efficiency and model complexity. The quantitative results are summarized in Table 3.

**Table 3:** Ablation study of waveMamba-YOLO under different lighting conditions.

Method	CHDWT	GLaM	LWGA	Normal	Low-light	Over-light	Uneven	FPS	Params
YOLOv11n				49.5	42.1	44.3	40.8	394	2.58
YOLOv11n+C	✓			50.5	46.0	46.8	43.9	380	2.65
YOLOv11n+G		✓		50.6	44.8	47.0	43.5	310	2.80
YOLOv11n+L			✓	51.2	45.4	47.2	46.0	347	2.77
YOLOv11n+C + G	✓	✓		50.9	47.1	48.0	45.0	290	3.05
YOLOv11n+C + L	✓		✓	51.4	47.9	48.3	46.7	305	3.02
WaveMamba-YOLO	✓	✓	✓	51.7	48.4	49.0	47.1	268	3.50

As shown in Table 3, each individual module improves detection performance over the YOLOv11n baseline across all illumination conditions. The CHDWT module achieves the most notable improvement under low-light conditions, increasing mAP_50_ from 42.1% to 46.0%. The GLaM module yields the largest single-module gain under over-light conditions (44.3% to 47.0%), while the LWGA module provides the strongest improvement under uneven illumination (40.8% to 46.0%). These results indicate that the three modules enhance robustness to illumination variation from complementary perspectives.

When the modules are combined, further performance gains are observed. The integration of CHDWT and LWGA improves mAP_50_ to 51.4% and significantly enhances robustness under challenging lighting conditions. The full WaveMamba-YOLO model achieves the best overall performance across all illumination settings, reaching 51.7% mAP_50_ under normal illumination and 47.1% under uneven illumination, while maintaining real-time inference speed of 268 FPS with only 3.5M parameters. Notably, the performance gap between normal and uneven illumination is reduced from 8.7 percentage points in the baseline to 4.6 percentage points, demonstrating improved illumination robustness.

### Comparative evaluation of wavemamba-YOLO and mainstream detectors on steel surface defect detection

To comprehensively evaluate the proposed WaveMamba-YOLO in terms of detection accuracy and generalization capability, we performed a series of comparative experiments against several mainstream real-time object detection models, including YOLOv12n [48], YOLOv8n [49], YOLOv5n [50], YOLOv11n [38], and two RT-DETR [51] variants. All models were trained and tested under the same experimental settings on the Severstal Steel Defect dataset, with a fixed input resolution of 640×640 to ensure fair comparison.

As detailed in Table 4, WaveMamba-YOLO outperforms all compared models across key evaluation metrics. It achieves the highest mAP50 (51.70%), mAP50–95 (23.90%), Recall (51.40%), and Precision (58.60%), while maintaining a compact parameter count of only 3.2M. In particular, compared to the baseline YOLOv11n, it delivers a notable improvement of +2.2% in mAP50 and +3.4% in Precision, demonstrating the efficacy of the proposed CHDWT, GLaM, and LWGA modules. For RT-DETR-X, while it shows a relatively competitive mAP50 (49.30%), multi-dimensional comparison reveals its limitations: its mAP50–95 (21.20%) is 2.7% lower than WaveMamba-YOLO, Recall (48.10%) is 3.3% lower, and it has 12.8× more parameters (41M vs. 3.2M) — leading to a 63% slower inference speed (18 FPS vs. 49 FPS for WaveMamba-YOLO, tested on an NVIDIA Jetson AGX edge device). These metrics collectively indicate that RT-DETR-X lacks practical superiority in accuracy, efficiency, and deployment feasibility for industrial scenarios.

**Table 4:** Benchmarking detection performance across steel defect detection models.

Method	Size	mAP50	mAP50-95	Recall	Precision	parameters
YOLOv11n [38]	640 x 640	49.50	22.70	48.80	55.20	2.6
YOLOv12n [48]	640 x 640	48.30	22.10	47.10	55.80	2.6
YOLOv8n [49]	640 x 640	49.10	23.00	50.10	52.20	3
YOLOv5n [50]	640 x 640	41.00	17.50	41.10	48.10	2.5
RT-DETR-I [51]	640 x 640	39.10	17.00	43.50	42.60	32
RT-DETR-X [51]	640 x 640	49.30	22.90	50.10	55.60	41
WaveMamba-YOLO	640 x 640	51.70	23.90	51.40	58.60	3.2

While WaveMamba-YOLO introduces a moderate increase in model size compared to YOLOv11n, the total parameter count remains well within the capacity of most edge and industrial devices, ensuring broad deployment feasibility. These results highlight the superior balance of accuracy and efficiency achieved by WaveMamba-YOLO.

**Fig 13:**
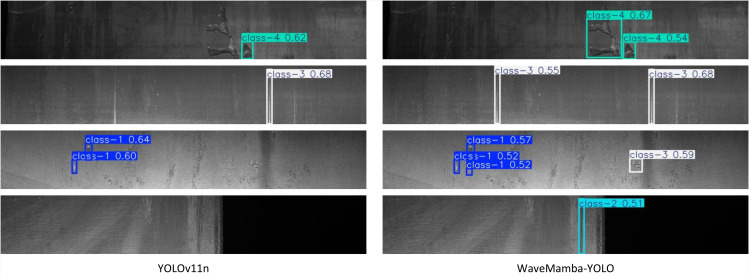
Visual Comparison of Defect Detection Performance Between YOLOv11n and WaveMamba-YOLO.

Fig 13 illustrates several qualitative comparisons that further highlight the advantages of WaveMamba-YOLO in localizing defects under challenging conditions. In the first row, WaveMamba-YOLO successfully detects two adjacent class-4 defects with high confidence scores (0.67 and 0.54), whereas YOLOv11n produces only a single low-confidence box (0.62), missing a portion of the defect region. In the second row, both models identify class-3 defects, but WaveMamba-YOLO yields tighter, less redundant bounding boxes. In the third example, WaveMamba-YOLO accurately detects three class-1 defects with stable predictions, while YOLOv11n struggles with duplicate or misaligned outputs. Notably, in the final case, YOLOv11n fails to detect the class-2 defect entirely, whereas WaveMamba-YOLO provides a precise detection with a confidence score of 0.51, underscoring its superior sensitivity to subtle or edge-localized anomalies. These results demonstrate the strong detection capability and generalization performance of WaveMamba-YOLO across varied defect types, including small-scale pits, elongated cracks, and large-area inclusions. The improvements can be attributed to the synergistic design of the CHDWT module for frequency-preserving downsampling, the GLaM module for direction-aware long-range dependency modeling, and the LWGA module for scale-specific attention refinement. Together, these innovations enable WaveMamba-YOLO to deliver high-accuracy, high-efficiency surface defect detection, making it well-suited for real-time quality inspection in industrial production lines.

### Performance evaluation on NEU-DET and GC10-DET steel surface defect benchmarks

To further assess the generalization capability and robustness of the proposed WaveMamba-YOLO, comparative experiments were conducted on two widely used industrial defect benchmarks, namely NEU-DET and GC10-DET. The NEU-DET dataset consists of six representative steel surface defect categories, including cracking, inclusion, patches, pitted surface, rolling scale, and scratches, which primarily reflect common texture degradations under relatively controlled acquisition conditions. In contrast, the GC10-DET dataset is a practical industrial benchmark collected from real steel sheet production lines, comprising 3570 grayscale images across ten defect categories, such as low-contrast water spots, oil stains, silk spots, and waist folds. Due to its realistic imaging environment, severe class imbalance, and diverse defect morphologies—many of which exhibit small spatial extent or irregular structures—GC10-DET poses substantially greater challenges to feature discrimination, scale adaptability, and model robustness. As such, it serves as an effective testbed for evaluating model generalization on rare and complex defect patterns.

For a fair comparison, all competing detectors were trained and evaluated under identical experimental settings, with the input resolution fixed at 640×640. As summarized in Table 5, WaveMamba-YOLO achieves the best overall detection performance on both datasets. On NEU-DET, it attains the highest mAP@0.5 of 77.70%, surpassing YOLOv11n, YOLOv12n, YOLOv8n, and YOLOv5n, while significantly outperforming transformer-based RT-DETR variants by a large margin.

**Table 5:** Performance comparison on NEU-DET and GC10-DET datasets. All models are evaluated under 640 × 640 resolution.

Method	Size	mAP@0.5		mAP@0.5:0.95		Recall		Precision		Params(M)	FLOPs(G)
		NEU	GC10	NEU	GC10	NEU	GC10	NEU	GC10		
YOLOv11n [38]	640 × 640	76.50	62.10	43.00	30.30	73.30	56.40	68.80	68.00	2.58	6.5
YOLOv12n [48]	640 × 640	74.70	62.70	40.70	29.80	70.10	55.80	67.80	67.20	2.55	6.5
YOLOv8n [49]	640 × 640	75.50	61.80	40.40	29.50	66.70	54.90	73.20	69.00	3.01	8.2
YOLOv5n [50]	640 × 640	76.00	62.00	39.60	29.20	68.30	54.10	70.00	66.50	2.51	7.2
RT-DETR-l [51]	640 × 640	51.70	50.80	25.50	22.80	49.40	46.50	51.50	60.00	32.83	108.0
RT-DETR-x [51]	640 × 640	49.30	49.10	22.90	21.50	50.10	45.20	55.60	61.30	67.32	232.4
**Ours**	640 × 640	**77.70**	**63.80**	**43.50**	**30.60**	**74.50**	**55.10**	**74.10**	**69.70**	3.5	7.0

More importantly, a consistent performance advantage is observed on the more challenging GC10-DET benchmark. WaveMamba-YOLO reaches 63.80% mAP@0.5 and 30.60% mAP@0.5:0.95, outperforming all lightweight YOLO baselines. Given the presence of numerous rare defect classes with small-scale or irregular morphological characteristics in GC10-DET, these results indicate enhanced robustness to class imbalance and improved multi-scale adaptability.

vIn addition, WaveMamba-YOLO maintains superior recall across both datasets, suggesting more stable defect localization and fewer missed detections under complex industrial conditions. Despite these performance gains, the proposed model preserves a lightweight architecture with only 3.5M parameters and 7.0 GFLOPs, remaining comparable to mainstream lightweight YOLO models while being substantially more efficient than RT-DETR variants. Overall, these results demonstrate that WaveMamba-YOLO achieves a favorable balance between accuracy, robustness, and computational efficiency, supporting its suitability for real-world industrial inspection scenarios.

**Fig 14:**
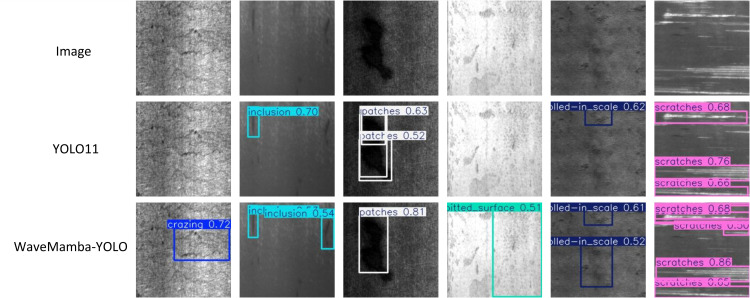
Visual comparison of YOLOv11n and WaveMamba-YOLO on NEU-DET.

Fig 14 further provides qualitative comparisons between YOLOv11n and WaveMamba-YOLO in representative samples. WaveMamba-YOLO shows clear advantages in accurately localizing small-scale defects (e.g., inclusions and scratches) and providing tighter bounding boxes with higher confidence scores. For example, in the first case, YOLOv11n fails to detect a crazing defect, while WaveMamba-YOLO successfully identifies it with a confidence of 0.72. Similarly, for inclusions and pitted surface defects, WaveMamba-YOLO produces more complete and stable detections, while YOLOv11n outputs redundant or misaligned boxes. These improvements highlight the contributions of the CHDWT, GLaM, and LWGA modules in enhancing the representation of features across scales and defect categories.

Overall, the results on NEU-DET confirm that WaveMamba-YOLO not only performs well on large-scale benchmarks such as the Severstal dataset but also generalizes effectively to smaller yet challenging industrial datasets. This robustness underscores its potential for practical deployment in real-world steel surface quality inspection.

## Conclusion

In this work, we presented WaveMamba-YOLO, a novel real-time detection architecture tailored for steel surface inspection. By incorporating three complementary modules—CHDWT for frequency-preserving downsampling, GLaM for direction-aware global-local context modeling, and LWGA for scale-adaptive attention refinement—the model achieves significant gains in precision, recall, and robustness. On the Severstal dataset, WaveMamba-YOLO attained 51.70% mAP@0.5 with only 3.2M parameters, outperforming state-of-the-art baselines while maintaining lightweight efficiency. Additional experiments on NEU-DET further validated its generalization capability across diverse defect types and scales.

Although WaveMamba-YOLO introduces slightly more parameters than conventional lightweight detectors, its performance advantages make it highly practical for real-time deployment in industrial inspection lines. Future work will focus on model compression and knowledge distillation to reduce complexity, as well as extending evaluations to cross-domain industrial datasets for broader applicability. Overall, WaveMamba-YOLO provides a scalable, accurate, and efficient solution for next-generation intelligent quality control systems.
